# Fabricating a Novel Intragranular Microstructure for Al_2_O_3_/GdAlO_3_ Ceramic Composites

**DOI:** 10.3390/ma11101879

**Published:** 2018-10-01

**Authors:** Shuai Sun, Qiang Xu

**Affiliations:** 1 Department of Materials, North China University of Technology, Beijing 100144, China; 2 National Key Laboratory of Science and Technology on Materials Under Shock and Impact, Beijing Institute of Technology, Beijing 100081, China; 3120100430@bit.edu.cn

**Keywords:** ceramic nanocomposite, spark plasma sintering, Al_2_O_3_, GdAlO_3_, intragranular structures

## Abstract

In order to make the embryonic form of intragranular structure, the Al_2_O_3_/GdAlO_3_ system was selected due to its excellent mechanical properties. Gd_2_O_3_ and Al(NO_3_)_3_·9H_2_O were used as the starting materials. A co-precipitation method was used for the preparation of fine ceramics and applied to synthesize the nano-powder of GdAlO_3_ firstly. Then, the nano-powder of GdAlO_3_ was mixed with the precipitates by the second co-precipitation method. After drying and calcination, the compound powder with eutectic composition (77 mol % Al^3+^—23 mol % Gd^3+^) was fast sintered by using the spark plasma sintering technique. The results revealed that the phases of the sintered samples were Al_2_O_3_ and GdAlO_3_. The phases showed a homogeneous and interlaced distribution. All the matrix grains were submicron. The sizes of the intragranular structures were between 50 nm and 150 nm. Therefore, the intragranular structure displayed a novel mixture of nanometer–submicron and submicron–submicron types. The different intragranular structures all changed the fracture modes of Al_2_O_3_ grains from intergranular fracture to transgranular fracture.

## 1. Introduction

Compared with traditional ceramics, ceramic nanocomposites have better properties of fracture strength, fracture toughness, creep resistance, and wear resistance [1]. Niihara thought ceramic nanocomposites could be divided into three categories: intragranular nanocomposite, intergranular nanocomposite, and nano/nano composite [2]. The intragranular microstructure is unique as one nanophase exists inside the matrix grain of the other phase. Therefore, the unique microstructure enables the nanocomposite ceramic to draw much attention in the research field.

Nowadays, the main nanocomposite ceramics in the non-rare-earth system are Al_2_O_3_/SiC(n), Si_3_N_4_/SiC(n), and ZrO_2_/Al_2_O_3_(n) (n stands for the nanoparticles in the above chemical compounds) et al. [3,4]. While, for the Al_2_O_3_/Re_3_Al_5_O_12_ (Re stands for the rare-earth elements) system, the Al_2_O_3_/Y_3_Al_5_O_12_ (YAG) composite ceramic is mainly focused on [5,6]. Liquid-coating method, reaction-sintering process, compound-powder method, and suspension-disperse-mixing method are the main synthetic methods to fabricate the intragranular microstructures [7]. The matrix grains with micron sizes are reported in the above mentioned systems. For the Al_2_O_3_/GdAlO_3_ system, excellent flexural strength and thermal stability at high temperature have been reported. Waku et al. found the Al_2_O_3_/GdAlO_3_ system displayed plastic deformation at 1873 K owing to dislocation motion, as in metals [8]. Ohashi et al. investigated the microstructures and orientation relationships of the Al_2_O_3_/GdAlO_3_ eutectic fibers fabricated by the micro-pulling down method [9]. Ma et al. prepared the Al_2_O_3_/GdAlO_3_ directionally solidified eutectic ceramics by laser floating zone melting process and studied the effects of composition and solidification rate on the microstructure of growth striations [10]. However, Al_2_O_3_/GdAlO_3_ composite ceramic with the novel intragranular microstructure remains unknown.

Eutectic chemical composition (77 mol % Al^3+^—23 mol % Gd^3+^) will enable the volume fractions of the two phases of the Al_2_O_3_/GdAlO_3_ composite ceramic to be close. Then, the fine microstructure can be expected due to the much higher volume fraction of the second phase (~50 vol %). However, the increase of the volume fraction of the second phase will suppress the formation of the intragranular microstructure [11,12]. To attempt to solve this contradiction, the nano-powder of GdAlO_3_ was synthesized firstly. Then, the nano-powder of GdAlO_3_ was mixed with the precursors synthesized via co-precipitation method.

Meanwhile, spark plasma sintering (SPS) technique that was assisted by electric field was applied to fabricate the expected novel microstructure. Compared with hot pressed sintering, the spark plasma sintering technique can achieve rapid sintering densification for the ceramic powders in a few minutes at lower temperatures [13,14]. It has often been used to prepare the advanced and fine ceramic composites. The features of the microstructures of the composite ceramic were analyzed and their effect to fracture mode was also explored.

## 2. Materials and Methods

Gadolinium oxide (Gd_2_O_3_, 99.99% in purity, Rare-Chem hi-tech co., ltd., Huizhou, China) and aluminium nitrate (Al(NO_3_)_3_·9H_2_O, 99.9% in purity, Sinopharm Chemical Reagent Co., Ltd., Shanghai, China) were used as the starting materials. The d_50_ of Al(NO_3_)_3_∙9H_2_O powder was 10 μm and the d_50_ of Gd_2_O_3_ powder was 3 μm. Preparation process of GdAlO_3_ was marked as routine 1. The process of routine 1 was as follows. Al(NO_3_)_3_·9H_2_O and Gd_2_O_3_ were weighed according to 1:1 of the stoichiometric ratio for Al and Gd elements, respectively. The powder of Al(NO_3_)_3_·9H_2_O was dissolved in deionized water. The chemical reaction is listed below.

Al(NO_3_)_3_·9H_2_O = Al(NO_3_)_3_ + 9H_2_O (1)

Then, Gd_2_O_3_ was dissolved in nitric acid to produce Gd(NO_3_)_3_.

Gd_2_O_3_ + 6HNO_3_ = 2Gd(NO_3_)_3_ + 3H_2_O (2)

Al(NO_3_)_3_ and Gd(NO_3_)_3_ were mixed in the homogeneous aqueous solution. For the co-precipitation method, the above salt solution and ammonia were both added into distilled water simultaneously in the back titration while pH value was kept between 8 and 9 till the end of the titration. The reactions between the above nitrate solutions and the ammonia are as follows.

Al(NO_3_)_3_ + NH_3_·H_2_O = Al(OH)_3_ + NH_4_NO_3_ (3)

Gd(NO_3_)_3_ + NH_3_·H_2_O = Gd(OH)_3_ + NH_4_NO_3_ (4)

In order to avoid the aggregation of small particles, ultrasonic fibrations were used during the co-precipitation reaction. The power of the ultrasonic instrument (Kunshan Ultrasonic Instruments Co, Ltd KQ-100DB, Kunshan, China) was selected as 100 W and the ultrasonic frequency was selected as 40 kHz. Then, the gelatinous precipitate was filtered and washed several times with water and ethanol, respectively. After drying at 120 °C for 24 h, the precipitates were calcined in air for 2 h, at a temperature ranging from 800 to 1200 °C, at a heating rate of 10 °C/min and cooled with the furnace.

The mixture process (routine 2) was as follows. The starting materials of Al(NO_3_)_3_·9H_2_O and Gd_2_O_3_ were weighed to achieve the final eutectic ratio (77 mol % Al^3+^—23 mol % Gd^3+^). The amount of GdAlO_3_ powder was selected to represent 2 vol % of the ceramic composite. Considering the dispersion of the GdAlO_3_ powder and the precipitation reaction, the pH value was kept between 9 and 10 [15]. At the same time, the nanoparticles of GdAlO_3_ from routine 1 were added during the co-precipitation reaction for routine 2. In order to gain a well dispersion of GdAlO_3_ nanoparticles, the ultrasonic and mechanical agitation were used during the reaction. After drying and calcination at 1100 °C, the compound powder was loaded in the graphite mold.

According to phase diagram of Al_2_O_3_-Gd_2_O_3_ [16], the temperature for the coexistence of GdAlO_3_ and Al_2_O_3_ phases is near 1600 °C. Therefore, the spark plasma sintering (SPS-3.20-MK-V, Sumitomo Coal Mining Co., Ltd., Kyoto, Japan) was conducted at 1600 °C for 3 min at a heating rate of 100 °C/min, at the pressure maintaining of 30 MPa and cooled with the furnace.

Phases of the calcined powder and the sintered sample were identified by an X-ray diffractometer (XRD, RIGAKU D/Max-rB, Tokyo, Japan) with Cu Ka radiation (0.1542 nm). The accelerating voltage was 40 kV with a tube current of 40 mA. The morphology of the calcined powder and the microstructure of the composite ceramic were examined by using a scanning electron microscope (FE-SEM, S-4800, HITACHI, Tokyo, Japan).

## 3. Results and Discussion

### 3.1. XRD Patterns of the Powder and Sintered Sample

**Figure 1:**
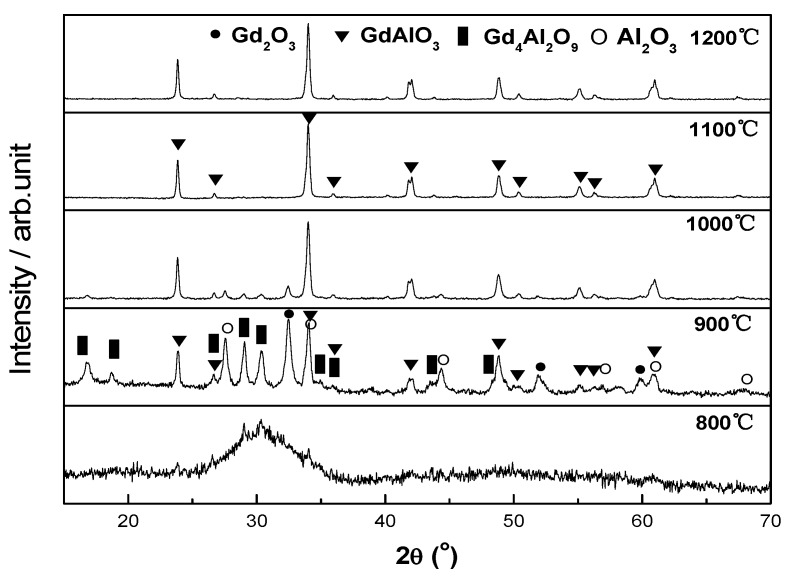
XRD patterns of the precursor powders of GdAlO_3_ calcined at different temperatures.

The XRD patterns of the precursor powders of GdAlO_3_ calcined at different temperatures are shown in Figure 1. As shown in Figure 1, there was no distinctive diffraction peak of the precursor powder calcined at 800 °C. When the calcination temperature got to 900 °C, the diffraction peaks were made up of GdAlO_3_, α-Al_2_O_3_, Gd_2_O_3_, and Gd_4_Al_2_O_9_. It can be known that hydroxide in the precursor underwent decomposition and the perovskite-type GdAlO_3_ began to crystallize. The reaction equations are shown as follows.

2Al(OH)_3_ = Al_2_O_3_ + 3H_2_O (5)

2Gd(OH)_3_ = Gd_2_O_3_ + 3H_2_O (6)

Gd_2_O_3_ + Al_2_O_3_ = 2GdAlO_3_ (7)

Further calcining at 1000 °C led to an increase in the intensity of the GdAlO_3_ peaks while the intensities of the diffraction peaks of α-Al_2_O_3_, Gd_2_O_3_, and Gd_4_Al_2_O_9_ decreased.

Gd_4_Al_2_O_9_ + 2Al_2_O_3_ = 4GdAlO_3_ (8)

When the calcination temperature reached up to 1100 °C, the phase of the calcined powder was only GdAlO_3_. It indicates that pure GdAlO_3_ whose precursor was synthesized via co-precipitation can be obtained after calcination at 1100 °C. For 1200 °C, the diffraction peaks of GdAlO_3_ had no change. Based on the kinetics and thermodynamics [17], when the stoichiometric ratios for Al and Gd were close, the transition phase of Gd_4_Al_2_O_9_ could be easily formed in the calcination process. When the stoichiometric ratio for Al and Gd was close to the eutectic ratio, the transition phase of Gd_3_Al_5_O_12_ could be easily formed in the calcination process [15].

**Figure 2:**
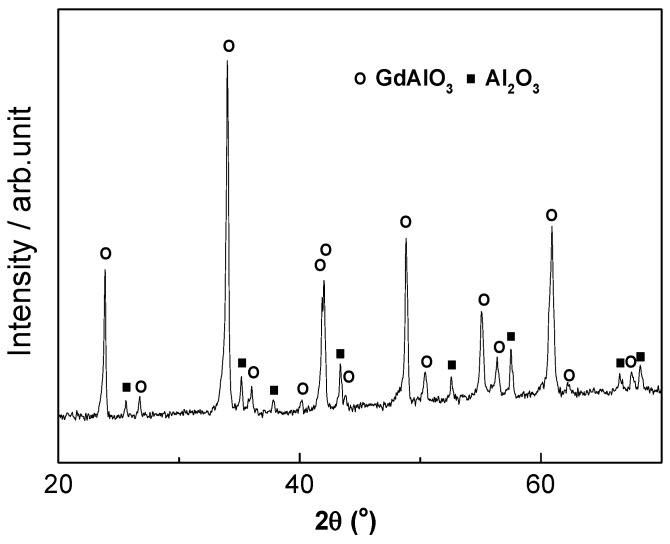
XRD pattern of sample with eutectic composition sintered by SPS.

The XRD patterns of the sample with eutectic composition sintered by SPS is shown in Figure 2. It shows that Al_2_O_3_/GdAlO_3_ composite ceramic is successfully fabricated and no impurity phase is found.

### 3.2. SEM Micrographs of the Powders

**Figure 3:**
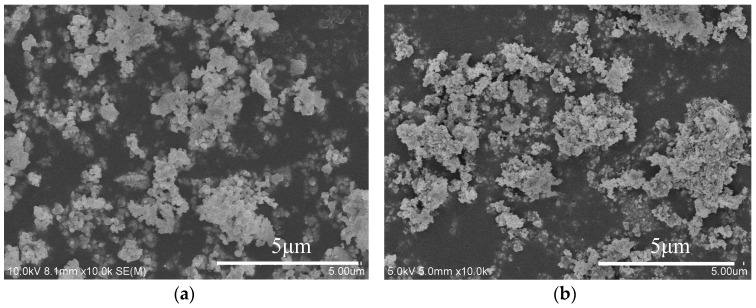
SEM micrographs of (**a**) the GdAlO_3_ powder (**b**) the compound powder synthesized by routine 2.

Figure 3a shows the SEM micrographs of the precursor powders calcined at 1100 °C. Primary nanoparticles are observed and they are slightly aggregated due to the drying and calcination processes. Figure 3b shows the SEM micrographs of compound powders prepared from routine 2.

### 3.3. Surface of Sintered Samples

**Figure 4:**
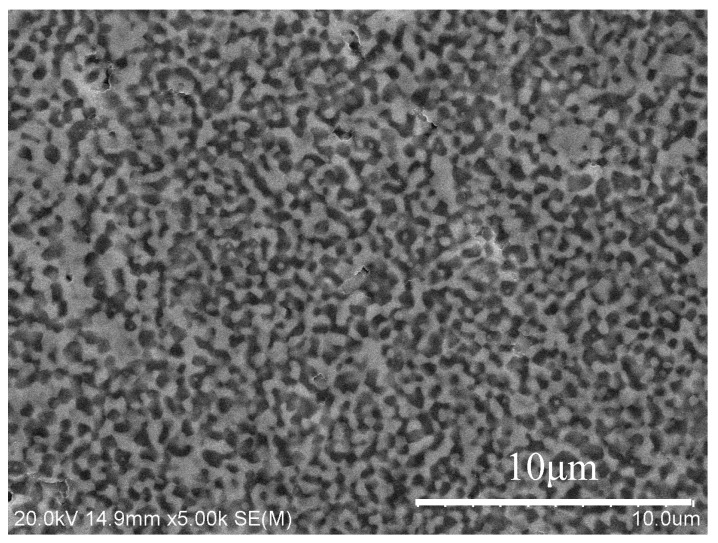
SEM micrograph of the polished surface of the SPS sintered sample.

The thickness of the sintered sample is about 2 mm and the diameter is about 10 mm. Figure 4 is the SEM micrograph of the polished surface of the sample sintered by SPS. It indicates that the microstructure of the ceramic composite is dense and made up of two phases. The average grain size of the microstructure is about 500 nm. According to the testing conditions, the brighter submicron phase is GdAlO_3_ and the darker submicron phase is α-Al_2_O_3_. It reveals that the homogeneous, interlaced and fine microstructure for Al_2_O_3_/GdAlO_3_ can be successfully prepared by the wet chemical process and the SPS technique.

### 3.4. Fracture Surface of Sintered Samples

**Figure 5:**
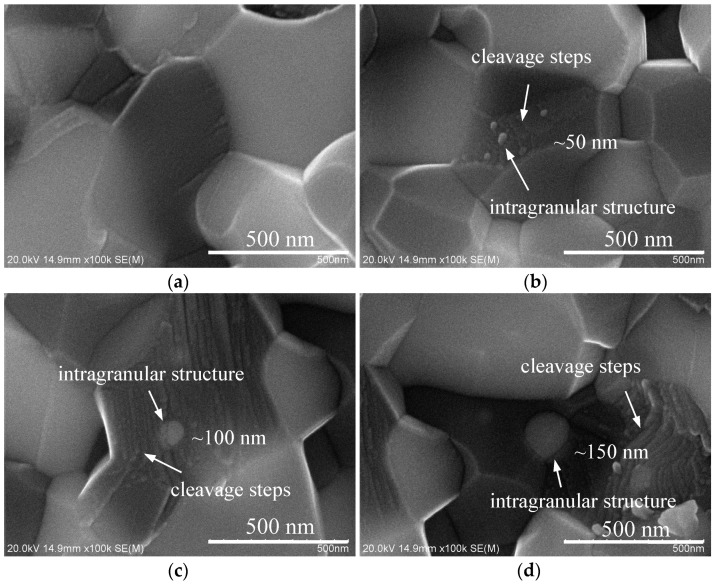
SEM micrographs of fracture morphologies (**a**) the Al_2_O_3_ grain without intragranular structure (**b**) the particle size of intragranular structure was about 50 nm (**c**) the particle size of intragranular structure was about 100 nm (**d**) the particle size of intragranular structure was about 150 nm.

Figure 5a shows the Al_2_O_3_ matrix grain without intragranular structure. It can be known that the two phases combines well and there is no impurity phase at the interfaces. Figure 5b shows there are several nano-particles (~50 nm) of GdAlO_3_ phase in the submicron matrix grain of Al_2_O_3_ phase to form the novel intragranular structure of the nanometre-submicron type. Moreover, the intragranular structures are both observed in the matrix grains of Al_2_O_3_ phase in Figure 5c,d. The average nano-particle sizes are about 100 and 150 nm in Figure 5c,d, respectively. The grain sizes of the intragranular structures in Figure 5c,d are relatively larger than those in Figure 5b. Furthermore, the amount of the nano-particles in Figure 5b is more than those in Figure 5c,d. Thus, the intragranular structures of the submicron–submicron type in Figure 5c,d are presented.

The formation of the intragranular structures is discussed as follows. The formation of the intragranular structures was associated with the chemical environment and the dispersion of GdAlO_3_ particles. When the GdAlO_3_ particles were dispersed and surrounded by more Al_2_O_3_ particles, it was beneficial to promote the diffusion and mass transfer for Al_2_O_3_ phase by using the spark plasma sintering process. It enabled the Al_2_O_3_ grains to have the relatively lower sintering temperature and faster migration velocity of crystal boundary. Then, with the enhanced growth of Al_2_O_3_ grains, the GdAlO_3_ nano-particles were probably swallowed up by the Al_2_O_3_ grain for the case of Figure 5b. When the GdAlO_3_ nano-particles were slightly aggregated, they were easily to form one larger grain during the sintering process for the cases of Figure 5c,d. When the GdAlO_3_ nano-particles were surrounded by other more GdAlO_3_ particles, the nanoparticles of GdAlO_3_ phase tended to grow into one larger submicron grain of GdAlO_3_ phase for the case of Figure 5a.

**Figure 6:**
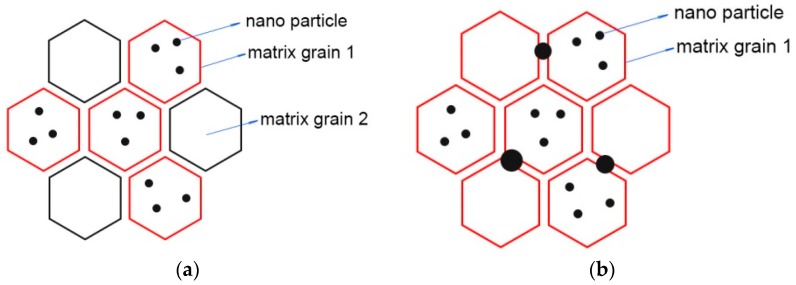
(**a**) Schematic diagram of this new microstructure (**b**) the traditional microstructure of the intragranular and intergranular types.

Based on the above discussion, the synthesized Al_2_O_3_/GdAlO_3_ ceramic composite has the novel microstructure as shown in Figure 6a. The novel microstructure is different from the traditional intragranular microstructure and intergranular microstructure. The volume fractions for Al_2_O_3_ phase and GdAlO_3_ phase are close and the well alternative distribution of the submicron matrix grains is formed. In the matrix grains of Al_2_O_3_ phase, nanoparticles with sizes of 50–150 nm in the intragranular microstructures are present. Up to now, the traditional intragranular microstructures of Figure 6b for Al_2_O_3_/YAG are mainly reported [18,19]. The common sizes of the matrix grains for the traditional intragranular microstructures are micron. The nanoparticles of YAG phase often locate in the matrix grains of Al_2_O_3_ phase and the larger particles of YAG phase locate at the grain boundares of Al_2_O_3_ phase, as shown in Figure 6b.

Generally, the fracture mode of Al_2_O_3_ grains is intergranular fracture [20,21]. It reveals that the intragranular structure tended to induce the transgranular fracture of Al_2_O_3_ grains in Figure 5d. Since the average coefficient of volume thermal expansion for GdAlO_3_ (~31.8 × 10^−6^/°C) is higher than that of Al_2_O_3_ (~21.9 × 10^−6^/°C) [17,22], the GdAlO_3_ nanoparticles in the intragranular structure may have larger volume contraction than that of Al_2_O_3_ matrix during the cooling process following the sintering densification. Thus, the GdAlO_3_ nanoparticles pulled the Al_2_O_3_ matrix for the GdAlO_3_/Al_2_O_3_ system at room temperature. The residual tensile stress field generated and tended to form microcracks near the interfaces of the intragranular structures. If the cracks nucleated near the intragranular structures, the stress field of the crack tip would interact with the residual tensile stress field and other microstructure defects as the external force loaded on the ceramic composite. When the shear stress value exceeded the cleavage strength of the ceramic composite, the cleavage crack would propagate along the specific crystal plane [23]. If the cracks nucleated far from the intragranular structures, the crack would be captured by the intragranular structure as it propagated [24]. Due to the interactions of residual stress and defects, the direction of crack propagation was deflected and the crack went through the Al_2_O_3_ matrix grain along the specific crystal plane. As described above, the cleavages formed for the cases of Figure 5b–d. Meanwhile, Figure 5b–d also present the cleavage patterns for the Al_2_O_3_ matrix grains. The cleavage patterns are mainly consisted of some parallel cleavage steps that are made up of the intersections of different cleavage planes.

## 4. Summary

In order to prepare the intragranular structures for Al_2_O_3_/GdAlO_3_ composite ceramic, GdAlO_3_ powder was synthesized at the calcination temperature of 1100 °C for 2 h by co-precipitation method. The nanocomposite ceramic of Al_2_O_3_/GdAlO_3_ with intragranular structures was successfully obtained by the chemical process and spark plasma sintering technique. The sizes of all the matrix grains were kept submicron. By the above techniques, the intragranular structure that contains GdAlO_3_ nanoparticles in the Al_2_O_3_ grains can be prepared for the Al_2_O_3_/GdAlO_3_ system, even suitable for Al_2_O_3_/ReAlO_3_ systems. The novel intragranular structures present two types: nanometre–submicron and submicron–submicron. The intragranular structures have changed the fracture mode of the Al_2_O_3_ phase and induced the transgranular fracture instead of intercrystalline fracture due to the residual stress. Furthermore, the features of cleavage in the Al_2_O_3_ grains display parallel cleavage steps.
